# Small molecules, enormous functions: potential approach for overcoming bottlenecks in embryogenic tissue induction and maintenance in conifers

**DOI:** 10.1093/hr/uhae180

**Published:** 2024-07-10

**Authors:** Tao Guo, Fen Bao, Yingming Fan, Jinfeng Zhang, Jian Zhao

**Affiliations:** State Key Laboratory of Efficient Production of Forest Resources, National Engineering Research Center of Tree Breeding and Ecological Restoration, Key Laboratory of Genetics and Breeding in Forest Trees and Ornamental Plants, Ministry of Education, The Tree and Ornamental Plant Breeding and Biotechnology Laboratory of National Forestry and Grassland Administration, College of Biological Sciences and Biotechnology, Beijing Forestry University, Beijing 100083, China; State Key Laboratory of Efficient Production of Forest Resources, National Engineering Research Center of Tree Breeding and Ecological Restoration, Key Laboratory of Genetics and Breeding in Forest Trees and Ornamental Plants, Ministry of Education, The Tree and Ornamental Plant Breeding and Biotechnology Laboratory of National Forestry and Grassland Administration, College of Biological Sciences and Biotechnology, Beijing Forestry University, Beijing 100083, China; State Key Laboratory of Efficient Production of Forest Resources, National Engineering Research Center of Tree Breeding and Ecological Restoration, Key Laboratory of Genetics and Breeding in Forest Trees and Ornamental Plants, Ministry of Education, The Tree and Ornamental Plant Breeding and Biotechnology Laboratory of National Forestry and Grassland Administration, College of Biological Sciences and Biotechnology, Beijing Forestry University, Beijing 100083, China; State Key Laboratory of Efficient Production of Forest Resources, National Engineering Research Center of Tree Breeding and Ecological Restoration, Key Laboratory of Genetics and Breeding in Forest Trees and Ornamental Plants, Ministry of Education, The Tree and Ornamental Plant Breeding and Biotechnology Laboratory of National Forestry and Grassland Administration, College of Biological Sciences and Biotechnology, Beijing Forestry University, Beijing 100083, China; State Key Laboratory of Efficient Production of Forest Resources, National Engineering Research Center of Tree Breeding and Ecological Restoration, Key Laboratory of Genetics and Breeding in Forest Trees and Ornamental Plants, Ministry of Education, The Tree and Ornamental Plant Breeding and Biotechnology Laboratory of National Forestry and Grassland Administration, College of Biological Sciences and Biotechnology, Beijing Forestry University, Beijing 100083, China

## Abstract

Somatic embryogenesis (SE) is not only the most effective method among various strategies for the asexual propagation of forest trees but also a basis for genetic improvement. However, some bottlenecks, such as the recalcitrance of initiation, the maintenance of embryogenic potential during proliferation and the low efficiency of maturation as well as high rate of abnormal embryo development remain unresolved. These bottlenecks refer to complex mechanisms, including transcriptional regulatory networks, epigenetic modifications and physiological conditions. In recent years, several small molecules utilized in animal stem cell research have exhibited positive effects on plant regeneration, including conifer species, which offers a potential novel approach to overcome the challenges associated with SE in conifers. In this review, we summarize the small molecules used in conifers, including redox substances, epigenetic regulatory inhibitors and other metabolism-related molecules, which overcome these difficulties without the use of genetic engineering. Moreover, this approach also has the advantages of dynamic reversibility, simple operation, and simultaneous regulation of multiple targets, which might be one of the best choices for optimizing plant regeneration systems including SE.

## Introduction

The cultivation of coniferous trees has the highest distinction in the world, boasting significant economic and ecological value [1]. Because conventional breeding is time consuming due to the long life cycle of conifers, somatic embryogenesis (SE), as the most efficient asexual propagation technique, holds significant value for the breeding and research of conifer species and is suitable for large-scale and automatic production, which provides great economic and ecological value [2]. Furthermore, SE combined with cryopreservation and molecular marker techniques could accelerate the breeding process and preserve germplasm resources [3]. With several breakthroughs in genome assembly [4, 5], which has provided good opportunities for molecular breeding and basic research in conifers, a stable asexual reproduction system (e.g., SE) has been urgently needed for the establishment of molecular tools, such as genome editing tools [6, 7]. Moreover, these molecular tools are also beneficial for revealing the mechanism of SE in conifers [8].

After more than 30 years of research, significant progress has been made on SE in conifer species, which have been applied for large-scale production and genetic improvement, particularly in *Pinus radiata* [9], *Pinus taeda* [10] and *Picea abies* [11]. However, there are still four technical challenges that currently hinder somatic embryogenesis technology for coniferous species:

(i) Low initiation frequency and limited sources of explants.
(ii) Embryogenic potential loss during long-term subculture.
(iii) Low maturation rates and high rate of abnormal embryo development.
(iv) Low rate of germination.

Over the past three decades, the optimization of SE in conifers has focused primarily on refining culture conditions [12] and selecting genotypes [13]; however, these challenges remain partially unresolved in numerous conifer species.

Somatic embryogenesis can be directly regulated by a related gene regulatory network, which further affects the signaling pathways and physiological states of cells. In recent years, significant progress has been made in the field of the genetic basis of SEs in plants, in which several genes, such as *LEC1/2* and *BBM*, regulate SEs correlated with plant growth regulators or other stresses [14]. Based on these findings, several studies using molecular tools that regulate gene expression to enhance somatic embryogenesis have been reported [8, 15, 16]. However, compared with those of angiosperms, functional studies of genes involved in somatic embryogenesis in conifers are still lacking. Furthermore, studies on SE utilizing angiosperm model systems (such as *Arabidopsis thaliana*) have revealed notable differences among conifers, particularly with respect to culture conditions, processes, and genes involved, due to the divergence between gymnosperms and angiosperms, which occurred approximately 300 million years ago [17]. For instance, *LEC2* might be absent in some conifers [18]. More importantly, genetic engineering technology is not an available option for most countries, and alternative methods to influence gene expression and physiological state need to be explored.

Notably, an approach that uses small chemical molecules to affect cell development and reprogramming has been successfully used in stem cell studies in mammals [19], reminding that it would also be possible to regulate gene expression or physiological state during SE using small molecules. Although the biological processes involved in animal studies are quite different from those involved in plant SE, the functions of small molecules, such as regulating epigenetic related enzymes, are mostly the same in both plants and mammals [20]. Several reports have noticed the chemical approach used in plant research [21], and several small molecules, such as the GSK3β inhibitor TDZD-8 [22] and the epigenetic inhibitor TSA [23], have been shown to promote plant cell reprogramming during somatic embryogenesis.

Although there are differences in terms of the technical protocols, developmental processes and specific gene functions in SE between conifers and angiosperms, the epigenetic regulation and endogenous metabolites, including reactive oxygen species (ROS) [24, 25], Ca^2+^ [26, 27], and endogenous hormones [28, 29] have similar functions in the regulation of SE, as all of them are involved in the transition of cell fate [30]. It provides a theoretical reference for the application of small molecules in the study of conifer SE. On the other hand, the approach using small molecules avoids the use of genetic tools while regulating gene expression and cell signaling. Surprisingly, a multitude of successful applications have been reported in plants, including conifers [29–31].

**Figure 1:**
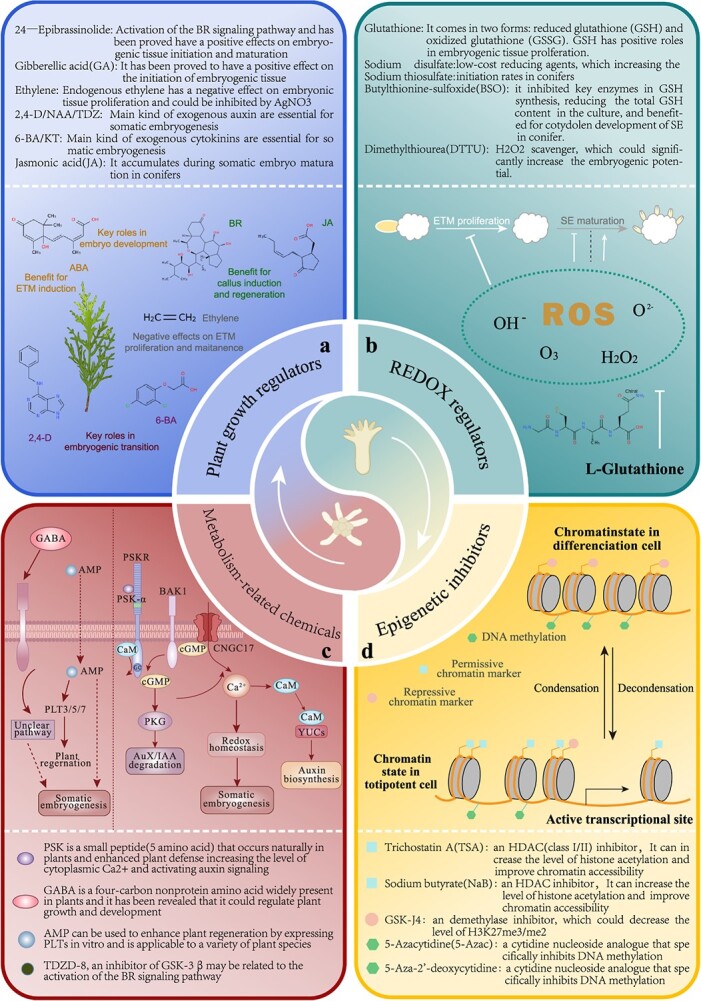
The main kinds of small molecules used in SE. **a** The main regulatory effects of major plant growth regulators on somatic embryogenesis. **b** The main role of reactive oxygen species during SE and the main kinds of reactive oxygen species regulatory substances. **c** Exogenous metabolic substances, such as peptides and their mechanism. **d** Introduction of epigenetic inhibitors to achieve embryogenic transition by regulating chromatin accessibility.

These studies suggest the potential value of small molecules in the SE of conifers. Notably, the approach for using small molecules in conifers includes that mimicking the hormonal, gene expression patterns, and physiological conditions found *in vivo* during immature zygotic embryo development or committing to alter the abnormal physiological or genetic states detected during SE to overcome related bottlenecks, which has the advantages of dynamic reversibility, simple operation, and simultaneous regulation of multiple targets. The small molecules summarized in this paper are introduced in Fig. 1 and mainly have the following characteristics:

(i) Chemicals with high cell penetrating and being easily absorbed.
(ii) Trace amounts (usually less than 1 mM) with dominant effects.
(iii) Molecular weight less than 900 Da.

In this review, we summarize the major challenges in the initiation and maintenance of embryogenic tissues (ETs) in conifers, focusing on physiological and epigenetic factors, and comprehensively summarize the works on small molecules such as REDOX regulators, epigenetic regulatory inhibitors and other metabolism-related molecules (Fig. 1a–c) to overcome these difficulties of SEs in conifers. Notably, plant growth regulators (Fig. 1a) are commonly used in SEs according to several methods [28, 31], so they are not discussed in detail. Furthermore, we discuss other small molecules involved in plant regeneration to discuss the potential of small-molecule approaches for SE in the future.

## The bottlenecks of ET initiation

The process of conifer somatic embryogenesis involves four key stages: induction and initiation of ET, proliferation of embryonic tissue, maturation of somatic embryos, and germination of somatic embryos [32]. ETs are commonly extruded from an immature zygotic embryo (IZE) and less from mature seeds, which are restricted by seasons. Furthermore, the initiation rate is highly dependent on the genotype, which is a time-consuming process for selecting superior families even if other factors affecting induction have been proven appropriately [1, 33].

Several studies have revealed that maternal influence is the reason why the efficiency of initiation and maturation are highly variable among different families. Research on the family hereditary impacts of *P. radiata* somatic embryogenesis has revealed a significant additive genetic influence on the initiation of ET, resulting in initiation rates ranging from 44% to 93% [34]. The effect of parent genotypes on the initiation of ET was evaluated in controlled crosses of seven *Pinus sylvestris* plants and the results revealed that the effect of the maternal parent was most pronounced at culture initiation, while the maternal effect decreased after 6 months in tissue culture; however, the effects of both parents were significant [35]. Furthermore, the additive effect of SE initiation on *P. taeda* was supported by the consistent results obtained from two independent experiments using different procedures with the same control-crossed immature seed [36]. These results indicate that a large improvement in ET initiation could be achieved in a predictable manner by large-scale selection of the most favorable female parent, although this process is also time-consuming. At present, the problem of low ET initiation efficiency is more serious in *Pinus* spp. than in *Picea* spp., as the initiation rate is less than 1% for some *Pinus* species, such as *Pinus pinea* [37] and *Pinus luchuensis* [38]. In contrast, the ET initiation efficiency of *Picea* could reach more than 10% [39, 40]. Although *P. radiata* [34], *P. taeda* L. [41], and *Pinus pinaster* Air [42] could reach the range of 40%–60%, these results were usually obtained from good crosses selected previously, while most of the genotypes still exhibited low efficiencies.

Compared with those explants used for SE in angiosperms, such as Arabidopsis, the explants used for somatic embryogenesis in conifers originate from a few single sources, among which immature embryos in the polyembryonic-cleavage stage are commonly used [43], as well as a few mature zygotic embryos [44, 45]. Generally, the initiation rate of mature zygotic embryos is significantly lower than that of immature zygotic embryos, although a few species exhibit a higher rate in the polycotydolenary embryo stage [46, 47]. Related studies have shown that embryos in the 16-cell stage [48] or polyembryonic-cleavage immature stage [49] can achieve greater initiation efficiency. As the embryo gradually matures, it becomes less sensitive to exogenous auxin and cytokinin stimulation and enters a developmental process; moreover, the initiation frequency decreases, regardless of the culture medium used [46]. This decrease in susceptibility may be the fundamental reason for the difficulty in achieving an embryonic response in these mature zygotic embryos [48].

Bonga (2010) summarized the characteristics of bottlenecks in ET initiation and somatic embryo differentiation in conifers as ‘recalcitrance’. Specifically, recalcitrance refers to the failure to produce the expected embryonic response through traditional methods such as adjusting the levels of inorganic salts, organic or inorganic nitrogen and auxin or cytokinin [48]. The recalcitrance observed in this study may be attributed to various factors, including differences in physiological or chromatin states affecting the genotype and developmental stage of the explants. These differences likely influence the responsiveness of embryonic transcription factors (TFs), such as *BBM* and *LEC1/LEC2*, which are the genetic basis for recalcitrance [50]. In addition, physiological states, such as the levels of endogenous hormones and redox states, are also important factors strongly related to ET initiation, as genotypes with lower endogenous IAA content are more likely to initiate ET during the polyembryonic cleavage stage of immature seeds in *Cunninghamia lanceolata* [29]. Research on the embryogenic development stages in loblolly pine revealed that an increase in glutathione (GSH) was maximal at the mid-development stage during the early and middle stages of embryo development and subsequently declined rapidly after the polycotydolenary stage [41]. Moreover, medium containing oxidation–reduction agents were found to increase the rate of embryonic tissue initiation [41]. In summary, it is difficult to overcome recalcitrance by simply adjusting the basal elements.

Notably, significant advancements have been achieved in the use of plant hormones, except auxin and cytokinin, and other chemical inhibitors to enhance the efficiency of ET initiation in conifers as well as in other species. As key regulatory substances of plant growth, plant growth regulators (PGRs) play a crucial role in somatic embryogenesis [31] (Fig. 1a). A lower concentration of abscisic acid (ABA) than that used at the mature stage can promote embryogenesis initiation [13] and positive effects of brassinolide (BR) [51] and ethylene [52] on conifers have also been reported. Recent studies showed that methyl jasmonate (MeJA) had a positive effect on promoting plant regeneration processes [53] and the endogenous jasmonic acid (JA) increased during embryo development of Norway spruce [28], indicating that it may contribute to SE in conifers.

**Table 1:** Comparison of the concentrations of PGRs added to ET-initiation and proliferation medium in coniferous species.

**Tree species**	**Initiation stage**		**Proliferation stage**		**Citation**
	**Auxin**	**Cytokinin**	**Auxin**	**Cytokinin**	
*Pinus radiata* (D. Don)	4.5 μM	2.7 μM	4.5 μM	2.7 μM	[9]
*Pinus taeda* L.	1.1 mg/L	0.45 mg/L	1.1 mg/L	0.45 mg/L	[62]
*Pinus oocarpa*	5 μM	4 μM	2.5 μM	2 μM	[49]
*Abies fraseri* [Pursh] Poir.	0.33 mg/L	1.1 mg/L	0 mg/L	1.1 mg/L	[63]
*Pinus heldreichii* Christ.	2 mg/L	0.2 mg/L	0.5 mg/L	0.05 mg/L	[64]
*Pinus pinea* L.	9 μM	4.5 μM	9 μM	4.5 μM	[37]
*Pinus sylvestris* L.	9 μM	4.5 μM	9 μM	4.5 μM	[65]
*Hybrid Larch* (*Larix* × *eurolepis* Henry)	2 mg/L	2 mg/L	0.5 mg/L	0.5 mg/L	[66]
*Larix principis-rupprechtii* Mayr.	2 mg/L	1 mg/L	0.2 mg/L	0.1 mg/L	[67]
*Abies cephalonica* Loud.	3.3 mg/L	2 mg/L	0 mg/L	0.2 mg/L	[68]
*Pinus pinaster* Ait*.*	13.5 μM	2.2 μM	0 μM	0 μM	[69]
*Abies bornmuelleriana*	0 mg/L	1.2 mg/L	0 mg/L	1.2 mg/L	_[_ _70__]_
*Pinus koraiensis* Sieb. et Zucc.	2.5 mg/L	1 mg/L	0.25 mg/L	0.1 mg/L	[61]
*Larix principis-rupprechtii* Mayr	2 mg/L	0.5 mg/L	0.2 mg/L	0.1 mg/L	[67]
*Pinus thunbergii* Parl*.*	2.2 mg/L	0.66 mg/L	1.125 mg/L	0.225 mg/L	_[_ _71__]_
*Pinus densiflora* Sieb. et Zucc.	2.2 mg/L	0.66 mg/L	1.125 mg/L	0.225 mg/L	[72]
*Picea balfouriana*	10.0 μM	13.5 μM	13.5 μM	13.5 μM	[73]
*Cryptomeria japonica* D. Don	10.0 μM	0 μM	0 μM	0 μM	[74]
*Picea abies*	10.0 μM	5.0 μM	10.0 μM	5.0 μM	[75]

Moreover, the achievement of cell totipotency is accompanied by epigenetic changes [54], as some epigenetic regulatory substances have also been found to be conducive to ET or embryogenic callus induction. Many studies have shown that the addition of the DNA methylation inhibitor 5-azacytidine (5Azac) has different effects at different stages of somatic embryogenesis [55]. In terms of histone modification, the histone deacetylase inhibitor TSA has been added to culture and was found to influence the somatic embryogenesis of *A. thaliana* [56] and *P. sylvestris* [57]. The potential effects of these epigenetic inhibitors on ET initiation as well as their basic mechanisms will be discussed in detail in ‘Epigenetic regulation of small molecules’.

## The bottlenecks in ET proliferation

The main problem at the ET proliferation stage in conifers is that long-term proliferation often leads to the loss of embryogenic potential and abnormal embryos at the maturation stage [58, 59]. This limits the application of SE technology in forest vegetative propagation and genetic improvement because elite cell lines can only be maintained for a few years, especially for *Pinus* species, which can be maintained for only a few months. Some studies have shown that this may be related to factors such as PGRs and subculture cycles used at the proliferation stage [60, 61].

Many established SE systems in conifers aim to retard the loss of ET embryogenic potential and soma clonal variation by reducing the concentration of PGRs during the proliferation stage, although embryogenic capacity can also be lost in hormone-free culture medium [58]. Table 1 presents recent PGR addition schemes for several conifer species with established SE systems. For instance, there was a positive effect of decreasing exogenous auxin to one-tenth of its original concentration during the proliferation stage in *Larix principis-rupprechtii* [67], although variations in effectiveness existed among different cell lines. For *Pinus oocarpa*, the proliferation rate could be increased, and a larger number of somatic embryos could be obtained by reducing the hormone concentration in the proliferation medium by half [49]. Notably, the protocols for some pines with high initiation efficiency, such as radiata pine and loblolly pine, involve maintaining consistent PGR concentrations during the proliferation and initiation stages to maintain a high proliferation rate. In general, the problem of loss of ET embryogenic capacity cannot be completely solved only by reducing the concentration of PGR as changes in the physiological states of tissues or cells are the underlying reasons for the loss of embryogenic potential [76, 77], which is not regulated but rather affected by exogenous hormones, such as auxin and cytokinin [39].

Compared to other PGRs, auxin and cytokinin play pivotal roles in the proliferation of ET and the preservation of ET differentiation capacity [40]. Cytokinin is a common PGR of somatic embryogenesis, for which most SE systems are required [78]. Moreover, some studies in recent years have reported that exogenous auxin has an impact on the physiology and genetics of SEs. As the most commonly used exogenous auxin in conifers, 2,4-dichlorophenoxyacetic acid (2,4-D) induces somatic embryogenesis more efficiently than natural auxin, which may be due to its auxin activity and stress-related responses [79]. These physiological changes do not occur independently of each other. Ethylene causes the oxidation of intracellular polyphenol compounds and membranes, which leads to the loss of the embryogenic response, while cell lines with low embryogenic potential were found to improve the efficiency of somatic embryo induction after the addition of ethylene inhibitors to the medium [80]. 2,4-D regulates somatic embryogenesis by affecting the overall level of DNA methylation, which requires the participation of S-adenosyl-l-methionine (SAM) [81]. Interestingly, SAM is not only an important methyl donor but also a precursor of ethylene biosynthesis [82].

Endogenous hormone levels, endogenous reactive oxygen species (ROS) activity, Ca^2+^ concentration, and epigenetic modifications are the key physiological and genetic markers affecting embryogenic potential. In long-term subcultured ETs, the levels of reactive oxygen species (ROS) increase, and the genes involved in ROS production are upregulated; moreover, accumulated hydrogen peroxide (H_2_O_2_) can promote intracellular Ca^2+^ accumulation [83] and regulate the state of embryonic tissues [27]. The addition of antioxidants could enhance the embryogenic potential. In addition, the structure and levels of endogenous hormones in embryonic tissue also change after long-term subculture. Disintegrated structure of ETs and irregularly shaped cells could be observed after long-term subculturing [84]. Higher indole-3-acetic acid (IAA)/ABA and GA (3)/ABA ratios are beneficial for maintaining embryogenic potential [84], while cell lines with high embryogenic potential usually have low levels of IAA [77]. However, another study reported that the endogenous level of IAA was significantly greater in ETs cultivated on medium supplemented with 2,4-D, which resulted in a greater rate of maturation than ETs subcultured on auxin-free medium [85]. Endogenous auxin plays an important role in maintaining embryogenic identity and somatic embryo development, and both embryonic genes and exogenous auxin can affect the level of endogenous auxin [86]. The correlation between IAA and embryogenic potential needs to be further studied in conifers. Epigenetic changes are also important. MiRNAs are regulated by the accumulation of zeatin-riboside (ZR) during long-term subculture, which affects embryogenic potential [73]. Moreover, DNA methylation is necessary for the maintenance of embryogenic potential. Changes in DNA methylation have been revealed in studies on *Araucaria angustifolia* [87]*, P. pinaster* [59]*,* and other plants, such as longan [88], oil palm [89], and soybean [90]. Furthermore, telomeres were found to shorten after long-term culture in *P. abies* [91]. In summary, the reasons for the physiological and genetic changes caused by exogenous hormones during long-term subculture leading to the loss of embryogenic potential are still not fully understood and further research was required.

Over the past three decades, other exogenous auxins, such as NAA, have replaced 2,4-D in several studies to optimize the protocol for ET proliferation [92]. Increased yields of somatic embryos were obtained using NAA and KT in the last subcultured medium before maturation in *P. radiata* [9]. Hazubska-Przybył *et al.* compared 2,4-D, NAA, and picloram during proliferation and found that there were no significant differences in maturation capacity between the two spruces [39]. Although there are some positive results in these studies, the problem of potential embryogenic loss remains unresolved.

Cryopreservation technology is currently the best solution for maintaining the embryogenic potential of ETs after long-term culture. Among the various methods of cryopreservation, slow-freezing method is the most common approach for conifer species [93], while the stepwise dehydration method is also feasible [94]. The scope of our review is focused on small molecules (chemical methods), so we do not discuss cryopreservation technology (physics methods) further.

## Potential positive effects of small chemical molecules

In recent years, an increasing number of genes associated with SE characteristics have been identified, and the molecular regulatory network of somatic embryogenesis has been revealed and improved, which has benefited from the rapid development of multiomics and genome editing technologies [8, 56, 95]. Due to the difficulties of genetic transformation in conifers and constraints in regulatory aspects, it is still difficult to improve the efficiency of somatic embryogenesis by regulating gene expression. However, as the genetic and physiological characteristics of somatic embryogenesis have gradually improved, a potential approach involving the use of small molecules for SE optimization has been developed. These molecules include reduction–oxidation (REDOX) regulators, epigenetic regulatory small molecules and small plant peptides that affect cell signaling pathways (Fig. 1). All of these factors combined with PGRs overcame some bottlenecks of SE in conifers as well as other species. In this section, we summarize the mechanisms and applications of these chemicals in ET initiation as well as proliferation and discuss their potential value for cell reprogramming and enhancing the embryogenic potential of conifers.

### REDOX modulators, with an emphasis on glutathione (GSH)

Many redox metabolic processes are involved in plant growth and development, with oxygen metabolites often referred to as ROS [96], which act as stress signals to deactivate intracellular stress responses, initiate posttranslational modifications (PTMs) [96] and affect gene expression [97]. During seed development, reduced glutathione (GSH)/glutathione disulfide (GSSG) and ascorbic acid (ASC, also called vitamin C)/dehydroascorbic acid (DHA) are the two main redox pairs that control the redox state, which is reduced at the beginning of embryonic development and is converted to an oxidized state at a later stage (Fig. 1b). The levels of ACS, an important reducing substance, peaked at the middle stage of embryo development in white spruce and then rapidly decreased, after which it was oxidized to DHA [98], indicating that early zygotic embryogenesis was in a reducing state and later transformed to an oxidizing state, which is an important reference for somatic embryogenesis. The addition of antioxidants at the initiation stage was conducive to improving the ET initiation rate, which increased in loblolly pine and Douglas fir in response to the addition of low-cost reducing agents, sodium disulfate and sodium thiosulfate [41]. On the other hand, an oxidation environment is needed to promote the normal development of embryos at the mature and germination stages of somatic embryos [99] (Fig. 1b).

GSH acts as an important antioxidant that protects cells from oxidative stress and plays roles in cell metabolism, antioxidant biochemistry, and redox homeostasis [100], and it was found that GSH could regulate SE in conifers. *Glutathione-S-transferase* (*GST*) genes tend to be highly expressed in embryonic tissues with high embryogenic potential in *Cryptomeria japonica* [101]. A series of studies on *Pinus koraiensis* revealed changes in GSH and GSSG among cell lines with different embryogenic capacities. Cell lines with strong ET proliferation capacity had a greater GSH:GSSG ratio, and the expression of *GSTs* was also generally upregulated, revealing the positive effect of GSH on proliferation in *P. koraiensis* [84, 102–104]. GSH also affects SE through regulating nucleic acid metabolism. Exogenous supplementation with GSH effectively enhanced embryonic production within the ET, leading to significant alterations in purine and pyrimidine nucleotide metabolism in proliferating cell lines [105].

An oxidation-type culture environment could be created by the addition of butylthionine-sulfoxide (BSO), which inhibits key enzymes involved in GSH synthesis, thereby reducing the total GSH content in the culture, and the effect on somatic embryogenesis might be triggered by the alteration of the REDOX state. Belmonte *et al.* (2007) showed that the conversion of total glutathione from the GSH state to the GSSG state helped to increase the number of somatic embryos in spruce, and the number of SE cotyledons under treatment with GSSG was greater than that in the control [106]. It was also found that during the maturation of white spruce somatic embryos, exogenous addition of BSO promoted the accumulation of stored substances (protein and starch) in SEs, while there were more vacuolated cells in the SEs of the control group [107].

Other intracellular redox-related substances, such as hydrogen peroxide (H_2_O_2_), are equally notable. According to a previous report on *Larix kaempferi* × *L. olgensis*, ETs with severe browning contain a relatively high level of H_2_O_2_ and a poor ability to form somatic embryos. The increase of H2O2 was greater in the ET which lost embryogenic potential after long-term cultured. Notably, treatment with N,N′-dimethylthiourea (DMTU) could partially recover the embryogenic potential [24, 27].

In conclusion, ROS usually play a negative role in the initiation and proliferation stages of ETs, while an oxidative environment is more necessary in the maturation and germination stages. The approach of small molecules according to the REDOX status at different stages or tissues of SE might play more important roles in optimizing SE.

### Epigenetic regulation of small molecules

**Figure 2:**
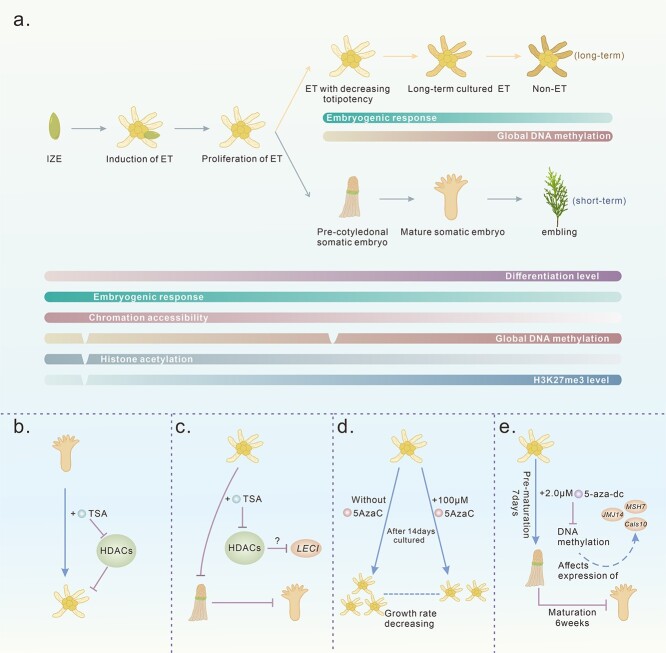
Levels of epigenetic modifications and embryogenic potential during somatic embryogenesis in conifers. **a** The underlying correlations between epigenetic modifications and SE. Different colors represent different modifications. The dark and light colors represent the strength and weakness of the signal or abilities, respectively. **b**–**e** Representative reports of EpIs affecting SE in conifers.

Because of the important role of epigenetic regulation in cell differentiation and dedifferentiation [108], several studies on epigenetics have revealed the regulatory mechanisms of DNA methylation, histone modification and miRNA in plant regeneration [109]. These epigenetic modifications not only regulate key transcription factors (TFs) that drive cell fate transition during regeneration but also directly affect the reprogramming of the overall transcriptome and chromatin states [110] (Fig. 1d). Specifically, from an epigenetic point of view, changes in overall DNA methylation and changes in chromatin conformation mediated by histone methylation and acetylation are common in embryo development [110]. We summarized the correlation between SEs and epigenetic modifications (Fig. 2).

Furthermore, epigenetic inhibitors (EpIs) have begun to be applied in research on plant regeneration. Here, we summarize several works reporting the use of EpIs in SE, including in conifers (Table 2), and provide comprehensive introductions in the following sections.

#### Histone acetylation

The level of histone acetylation changes dynamically during various regeneration processes and has been shown to play an important regulatory role in seed development, chromatin accessibility, and stress response [129]. Studies have shown that the functions of two sets of TFs (AUX/IAAs and ARFs), which are essential for the regulation of auxin signaling in plant cells, are related to two key regulatory enzymes involved in histone acetylation, HATs and HDACs [130]. HATs are known to be responsible for histone acetylation, while HDACs play a role in clearing acetyl groups from acetylated histones. During the regeneration process, acetylation of the promoter region of related genes can promote the induction and regeneration of calli in *Arabidopsis* [131, 132]. HATs/HDACs also regulate SE-related genes by catalytic (de)acetylation [122].

Histone acetylation can be reversed by small molecules. Although it is difficult to change the acetylation level of specific genes, cell reprogramming can be regulated by adjusting the overall acetylation level to achieve somatic embryogenesis and other regenerative processes. As previously mentioned, TSA or NaB, two kinds of histone deacetylase inhibitors, have been widely used in plant regeneration (Table 2). In medium supplemented with inhibitors, the efficiency of regeneration, such as callus formation [128], microspore embryogenesis [133], and somatic embryogenesis [121]could be improved. In addition, HDACs also regulate the homeostasis of ROS and IAA, which participate in the regulation of somatic embryogenesis [124].

Moreover, inhibitors of HDACs have a positive effect on overcoming the recalcitrance of somatic embryogenesis, as embryogenic callus can be induced from mature seeds, which is difficult in some species [23]. Interestingly, the initiation rate of ETs from cotyledonary embryos could be significantly increased in conifers, but it was still highly dependent on genotype, and it was also found that treatment with TSA caused derepression of *LEC* genes, which might be regulated by HDACs [57, 126] (Fig. 2b and c). As the *LEC *genes were derepressed in medium supplemented with TSA, the maturation and germination of ETs were arrested, and ETs continuously proliferated. Based on the above conclusions, it would be interesting to determine whether TSA can improve the initiation rate of immature zygotic embryos and restore embryogenic potential.

The induced concentration of TSA generally ranges from 1–10 μM, and it is critical to determine the optimal induced concentration. In addition, chemical reprogramming appears to be spatiotemporally specific, with different effects on explants of different species. In the process of leaf-induced regeneration, the addition of TSA reduced the callus induction rate. A similar situation also exists in conifers. After TSA induction of embryonic tissue, TSA continued to be added at the maturation stage, and it was found that the maturation efficiency of somatic embryos was reduced compared with that of the control [126].

#### Histone methylation

Lysine or arginine residues in the histone tail can be modified by monomethylation, dimethylation, or trimethylation to affect transcription by altering the local chromatin status. Unlike acetylation, the methylation of residues at different histone locations has different effects on the transcriptional regulation of genes. H3K27me3 and H3K9me3 have negative side effects on gene expression, while H3K4me3 and H3K36me3 are associated with transcriptional activation. H3K27me3 is an important type of transcriptional inhibitory posttranslational modification that promotes chromatin condensation and is mainly catalyzed by Polycomb repressive complex-2 (PRC2), as reviewed previously [134]. PRC2-mediated H3K27me3 plays a significant role in cell fate transition in plants [135]. Interestingly, studies have shown that H3K27me3 in *P. abies* is similar to that observed in other plant species and mainly accumulates in genic regions of the genome. Moreover, H3K27me3 levels in ETs were much lower than those in non-embryogenic tissues (non-ETs) and emblings but significantly increased after somatic embryo induction [95]. Another study of polycomb repressive complex 2 (PRC2) subunits revealed that some of these subunits are highly conserved in land plants [136]. These reports show that it is possible to regulate SE in conifers by regulating H3K27me3. GSK-J4, a demethylase inhibitor of H3K27me3, was found to significantly reduce the development of peach callus. However, no additional studies on the histone methylation-mediated regulation of somatic embryogenesis by inhibitors have been reported. Because the mechanism by which histone methylation is catalyzed is conserved in plants [137], another inhibitor, BIX-01294, which was used in microspore embryogenesis research, might be worth attention in the future [120].

**Table 2:** Summary of a less of the use of EpIs in embryogenesis.

**Species**	**Inhibitor used**	**Type**	**Concentration**	**Effects on embryogenesis**	**Reference**
*Arabidopsis thaliana*	5-Azacytidine (5-Azac)	DNA methylation	10 μM	It strongly inhibited the formation of somatic cell embryos and produced many non embryonic callus.	[111]
*Brassica napus*	5-AzaC	DNA methylation	2.5 μM	Somatic embryo induction increased, while long-term treatment decreased.	[112]
*Coffea canephora*	5-AzaC	DNA methylation	20 μM	5-AzaC added on day 21 after induction synchronized the embryogenic process but reduced the maturation of somatic embryos.	[113]
*Cocos nucifera* L*.*	5-AzaC	DNA methylation	15 μM and 20 μM	Pretreatments with 5-AzaC for 3 days significantly increased early somatic embryo formation.	[114]
*Cucurbita pepo* L.	5-AzaC	DNA methylation	12.3 μM	No significant effects on the efficiency of embryo proportion.	[115]
*Dimocarpus longan Lour*	5-AzaC	DNA methylation	20 μM	The embryogenic callus induction rate of pretreatment 3 days was higher than that of pretreatment 7 days, while the development of somatic cell embryos was blocked after pretreatment 7 days.	_[_ _88__]_
*Laris x eurolepis*	5-AzaC	DNA methylation	100 μM	It significantly reduced the relative growth rate of embryogenic cultures and embryogenic potential.	[115]
*Medicago truncatula*	5-AzaC	DNA methylation	100 μM	Production of somatic embryos and proliferation of non-embryogenic callus were decreased.	_[_ _116__]_
*Picea omorika*	5-AzaC	DNA methylation	12.3 μM	The total number of embryos developed in the subsequent transfer to the maturation medium was not significantly different, though DNA methylation decreased by 19%.	[117]
*Pinus pinaster*	5-AzaC	DNA methylation	10 and 15 μM	The highest amounts of somatic embryos were obtained at the 10 and 15 μM concentrations of 5-AzaC.	[59]
*Picea glauca*	5-aza-2′-deoxycytidine	DNA methylation	2 μM	The global DNA methylation level decreased on the prematuration cultured stage and the 1st week of maturation cultured.	[118]
*Theobroma cacao*	5-AzaC	DNA methylation	20 μM	Aged embryogenic callus after long-term cultured could recover embryogenic potential when treated with 5-AzaC.	[119]
*B. napus*	BIX-01294	histone methylation	0.5-5 μM	BIX-01294 promoted embryogenesis induction, while diminishing H3K9 methylation. DNA methylation reduced by short-term BIX-01294 treatment but long-term BIX-01294 treatments inhibited embryogenesis progression.	[120]
*A. thaliana*	Trichostatin A(TSA)	Histone acetylation	1.0 μM	Treatment with TSA, which is a chemical inhibitor of histone deacetylases, induced somatic embryogenesis without the exogenous application of auxin.	[121]
*A. thaliana*	TSA	Histone acetylation	1.0 μM	TSA treatment affected the expression level of the *LEC1, LEC2, FUS3*, and *MYB118* genes positively, implying a positive relationship between Hac and the expression level of these *TF*s	[122]
*A. thaliana*	4-Phenylbutyric acid(4-PBA)	Histone acetylation	20 μM；1 mM	4-PBA promotes the formation and regeneration of callus, not through inhibiting histone acetylation, but related to auxin response.	[123]
*D. longan*	Sodium butyrate (NaB)	Histone acetylation	10 mM	It reduced the proliferation and delayed the differentiation of embryogenic callus.	[124]
*Hordeum vulgare* L.	TSA	Histone acetylation	7.5 μM	TSA treatment with 7.5 μM had two times higher efficiency and productivity of plant regeneration.	[125]
*Picea abies*	TSA	Histone acetylation	0.1–10 μM	A less of embryogenic tissues was induced from zygotic embryos; however, the embryogenic potential decreases during germination.	[126]
*Pinus sylvestris*	TSA	Histone acetylation	0.1-10 μM	Same as abovementioned in *P. abies*.	[126]
*P. sylvestris*	TSA	Histone acetylation	10 μM	A less of embryogenic tissue was induced from approximately 70% of the cotyledonary embryos after treatment with TSA, which was depended on the genotype of explants (embryos).	[57]
*Vitis vinifera* L*.*	TSA/NaB	Histone acetylation	TSA:0.5-5 μM NaB:0.5–5 mM	These inhibitors resulted in an improvement of the embryogenic responses in grapevine.	[127]
*Triticum aestivum* L*.*	NaB	Histone acetylation	200 and 1000 μM	Appropriate addition of SB could increase the efficiency of embryogenic callus formation.	[128]

#### DNA methylation

DNA methylation is essential for plant development and is one of the most significant and intensively studied aspects of epigenetic regulation. In contrast to animal DNA methylation, DNA methylation in plants has three kinds of sequence contexts, CG, CHG, and CHH, which have different regulatory mechanisms and functions [138]. DNA methyloming is an important method for studying DNA methylation in plants. In *P. abies*, the DNA methylation levels of CG and CHG in Norway spruce were greater, and CHH methylation was lower than that in most other plants. Compared with those in needles, the levels of CG and CHG methylation and CHH methylation in ET-treated plants were lower than those in needle-treated plants [139]. However, the effects of different sequence contexts on somatic embryogenesis need to be further studied in conifers.

Many studies have shown that the global methylation level is correlated with SE in conifers. As we mentioned in Chapter 2, the global DNA methylation level affects the embryogenic potential of ETs after long-term subculture. Compared with that in medium without PGRs, the overall level of DNA methylation of ETs in *A. angustifolia* increased, and the embryogenic potential decreased after long-term proliferation [87]. Similar results were also found in *P. pinaster* [59]. In addition, it was found that non-ETs had higher methylation levels than ETs, and there was a difference in the conformation of the DNA between these tissues in *P. radiata* [140]. Another study showed that *Pinus nigra* had the lowest methylation levels in *cell lines with greater embryonic potential* [141]. The ability of mature somatic embryos to initiate embryonic tissue was also found to be correlated with DNA methylation in *Picea glauca* [142]. Moreover, DNA methylation is also involved in the effect of high-temperature stress on SE in conifers [143, 144].

Based on the above studies, embryogenic potential might be improved or restored by altering the overall level of DNA methylation. 5-Azac is a commonly used small molecule that inhibits DNA methylation and has different effects on SEs in plants (Table 2). After treatment with 5-Azac, the proliferation rate of fresh tissue decreased, and the maturation of somatic embryos slightly improved in response to aged ETs in *P. pinaster* [59], confirming that treatment with high concentrations (100 μM) inhibited proliferation [145] (Fig. 2d). In addition, another inhibitor, 5-aza-2′-deoxycytidine (5-aza-dc), was used in *P. glauca* (Fig. 2e), and the results showed that genes might be regulated by DNA methylation during somatic embryo development [118].

Although the use of small molecule inhibitors to regulate DNA methylation is simple, the impact on SEs may be difficult to predict. These inhibitors can reduce overall methylation levels; however, the methylation level of specific genes might be more important [146]. On the other hand, the functions of small molecules might differ greatly due to differences in concentration and period of treatment [55].

### Small peptides that affect metabolism

In this section, we introduce some small molecules (or peptides) related to cell metabolism and signaling and summarize their potential roles in SE, especially in conifers. We briefly introduce some recent reports that introduced a few novel SE molecules that have not yet been reported in conifers to provide potential approaches for researchers studying conifers.

#### γ-Aminobutyric acid (GABA)

As a four-carbon nonprotein amino acid, GABA is widely present in plants, and it has been revealed that it can regulate plant growth and development as a signaling molecule [147]. It is closely related to the TCA cycle and carbon and nitrogen metabolism, which might regulate SEs by interacting with phytohormones [148].

GABA has been used in studies of somatic embryogenesis. Studies on *Acca sellowiana* have shown that endogenous GABA reaches its highest concentration on day 9 and then decreases with fluctuations within 30 days [149]. The addition of exogenous GABA had a positive effect on the induction of somatic embryos and reduced the proportion of abnormal embryos [150].

Although there are few studies on the effect of GABA on SE in conifers, there is still evidence of its potential value. An analysis of the metabolite contents in the proliferation and maturation medium revealed that the GABA content was greater in the maturation medium, and the fluctuations in the endogenous GABA concentration during embryo development were similar to those in carrot medium [151], which could be partly explained by the demand for polyamines during embryo development [152]. Taken together, these findings imply the potential influence of these factors on SE in conifers.

#### Phytosulfokine (PSK)

PSK is a small peptide that occurs naturally in plants and enhances plant defense by increasing the level of cytoplasmic Ca^2+^ and activating auxin signaling [153]. PSK can also promote cell growth and proliferation by phosphorylating the glutamine synthetase GS2 [154]. In addition, PSK can enhance phytosulfokine receptor (PSKR) heterodimerization with somatic embryogenesis receptor-like kinases (SERKs) and activate downstream signaling pathways [155].

Since 2000, PSK has been found to be beneficial for SE in plants. Hanai, H. *et al.* first reported that PSK could promote the proliferation of embryogenic cells in carrots [156]. Since then, several positive effects of PSK on SE have been reported [157], including in conifers. PSK-α not only promotes the division of embryonic cells but also plays a significant role in the development of the suspensor [158]. In *C. japonica*, somatic embryo formation is stimulated, and cotyledons, hypocotyls, and roots are simultaneously sprouted [159]. In *C. lanceolata*, PSK not only increased SE efficiency but also overcame the bottleneck of establishing SEs in recalcitrant genotypes and effectively inhibited PEM browning during suspension culture by regulating ROS levels, which are important for ET initiation and proliferation [160]. Moreover, PSK has also been successfully used in microspore embryogenesis [161] and organogenesis [162] because of its strong functions and potential in plant regeneration. The mechanism of PSK in somatic embryogenesis needs to be further studied. Notably, PSK activated the PSKR-mediated phosphorylation pathway to promote calcium influx and increase cGMP levels (Fig. 1c), which might also explain the correlation between SE and PSK [163].

## Prospects and suggestions

In this review, we summarize in detail the current research progress in the field of improving SE systems by small molecules, focusing on ET initiation and proliferation. In addition, there are some small molecules that we have not mentioned in this paper as they have not been used in conifers.

With TDZD-8 as the primary representative, a GSK3β inhibitor that can suppress the activity of glycogen synthase kinase 3 (GSK3) which also been identified in plants and has a similar structure in conifers, indicating a potential function in somatic embryogenesis [164]. These inhibitors increase the efficiency of SE and microspore embryogenesis in *Quercus suber*, which may be related to the activation of the BR signaling pathway to regulate endogenous auxin and related genes in plants [22]. Because BRs are also important in conifers [165], TDZD-8 might also have potential in conifers. 4-Phenylbutyric acid, a histone deacetylase inhibitor, has recently been found to promote callus formation not by affecting the degree of histone acetylation but through its auxin activity, which might also have potential for the initiation of ET as a supplement to exogenous auxin in conifers [123]. Furthermore, AMP, a small molecule involved in energy metabolism, could enhance shoot regeneration and SE efficiency in *Arabidopsis* by promoting the expression of *PLT3/5/7* [166]. The promotional effects of cytokinin oxidase/dehydrogenase, exogenous CaCl_2_, the Ca^2+^ channel ionophore A23187, arabinogalactan proteins, and other substances on somatic embryogenesis have also been reported [26, 167, 168].

The fundamental principle underlying the small-molecule approach discussed in this review is to correlate the functions of small molecules with the corresponding physiological or genetic changes occurring in ETs or cells during SE to establish a conducive environment for SE. Consequently, a comprehensive understanding of conifer-related challenges is imperative for the application of small molecules. Focusing on the initiation and proliferation stages of SEs in conifers, we propose two crucial issues that hold promise for resolution through a small molecule approach:

(i) The mechanisms underlying the failure of the initiation of embryonic tissues by mature zygotic embryos or even vegetative tissues, such as needles or buds, have been confirmed.
(ii) In-depth analysis of physiological and genetic changes in the decline of embryogenic potential in ETs in conifers.

These problems can be solved by the use of novel omics techniques that have been widely used in plant research, such as ATAC-seq [169], CUT&Tag [170], single-cell RNA-seq [171], and WGBS [139]. With the complete annotation of the conifer genome publicly available [4], these techniques could be widely used, which could help us to reveal a more comprehensive understanding of the genetic regulatory network of SEs in conifers and the similarities and differences between conifers and other plants, which is also essential for small-molecule approaches.

In summary, the use of exogenous small molecular chemicals to improve somatic embryogenesis has broad potential, especially in conifers, and continuous attention and research on this topic are highly recommended.
